# Diagnosis and Management of Posterior Cortical Atrophy

**DOI:** 10.1007/s11940-022-00745-0

**Published:** 2023-02-08

**Authors:** Keir X. X. Yong, Jonathan Graff-Radford, Samrah Ahmed, Marianne Chapleau, Rik Ossenkoppele, Deepti Putcha, Gil D. Rabinovici, Aida Suarez-Gonzalez, Jonathan M. Schott, Sebastian Crutch, Emma Harding

**Affiliations:** 1 Dementia Research Centre, UCL Queen Square Institute of Neurology, Box 16, Queen Square, London, WC1N 3BG UK; 2 Department of Neurology, Mayo Clinic, Rochester, MN USA; 3 Nuffield Department of Clinical Neuroscience, University of Oxford, Oxford, UK; 4 School of Psychology and Clinical Language Sciences, University of Reading, Reading, Berkshire UK; 5 Memory and Aging Center, University of California San Francisco, San Francisco, CA USA; 6 Alzheimer Center Amsterdam, Department of Neurology, Amsterdam Neuroscience, Amsterdam UMC, Amsterdam, Netherlands; 7 Clinical Memory Research Unit, Lund University, Lund, Sweden; 8 Frontotemporal Disorders Unit, Massachusetts General Hospital and Harvard Medical School, Boston, MA USA; 9 Department of Psychiatry, Massachusetts General Hospital and Harvard Medical School, Boston, MA USA; 10 Department of Neurology, Radiology, and Biomedical Imaging, University of California San Francisco, San Francisco, CA USA

**Keywords:** Posterior cortical atrophy, Alzheimer’s disease, Visual variant Alzheimer’s disease, Visual processing, Atypical Alzheimer’s disease, Treatment

## Abstract

**Purpose of review:**

The study aims to provide a summary of recent developments for diagnosing and managing posterior cortical atrophy (PCA). We present current efforts to improve PCA characterisation and recommendations regarding use of clinical, neuropsychological and biomarker methods in PCA diagnosis and management and highlight current knowledge gaps.

**Recent findings:**

Recent multi-centre consensus recommendations provide PCA criteria with implications for different management strategies (e.g. targeting clinical features and/or disease). Studies emphasise the preponderance of primary or co-existing Alzheimer’s disease (AD) pathology underpinning PCA. Evidence of approaches to manage PCA symptoms is largely derived from small studies.

**Summary:**

PCA diagnosis is frequently delayed, and people are likely to receive misdiagnoses of ocular or psychological conditions. Current treatment of PCA is symptomatic — pharmacological and non-pharmacological — and the use of most treatment options is based on small studies or expert opinion. Recommendations for non-pharmacological approaches include interdisciplinary management tailored to the PCA clinical profile — visual-spatial — rather than memory-led, predominantly young onset — and psychosocial implications. Whilst emerging disease-modifying treatments have not been tested in PCA, an accurate and timely diagnosis of PCA and determining underlying pathology is of increasing importance in the advent of disease-modifying therapies for AD and other albeit rare causes of PCA.

## Introduction

Posterior cortical atrophy (PCA) is a clinico-radiological syndrome characterised by the progressive loss of higher order visuospatial, visuoperceptual and other posterior cortical functions consistent with occipito-parietal and occipito-temporal atrophy. Core features of PCA include space and object perception deficits, elements of Balint syndrome (simultanagnosia, oculomotor apraxia, optic ataxia), constructional dyspraxia, environmental agnosia, dressing apraxia, alexia, elements of Gerstmann syndrome (acalculia, agraphia, left–right disorientation, finger agnosia), alexia and apraxia, with relative sparing of other cognitive domains [1] (Table 1A). Amongst neurodegenerative disorders, PCA tends to have a young onset presentation (83% with age at onset < 65 years) with patients affected as young as in their 40s or as old as in their 90s [2]. Whilst PCA (previously termed ‘Benson’s syndrome’) can be underpinned by other pathologies, retrospective and prospective neuropathological [3–5] and biomarker [6–8] studies have reported evidence of primary or co-existing AD pathology in > 75% of cases. This predominance is consistent with PCA being considered one of the major atypical AD phenotypes and designation as ‘visual variant-’, ‘biparietal-’ or ‘visual-spatial’ AD [9, 10••]. Limited estimates of the prevalence of PCA or visual-led AD based on specialist dementia clinics suggest that 8–13% of patients may present with visual- or praxis-predominant presentations [11, 12].

**Fig. 1:**
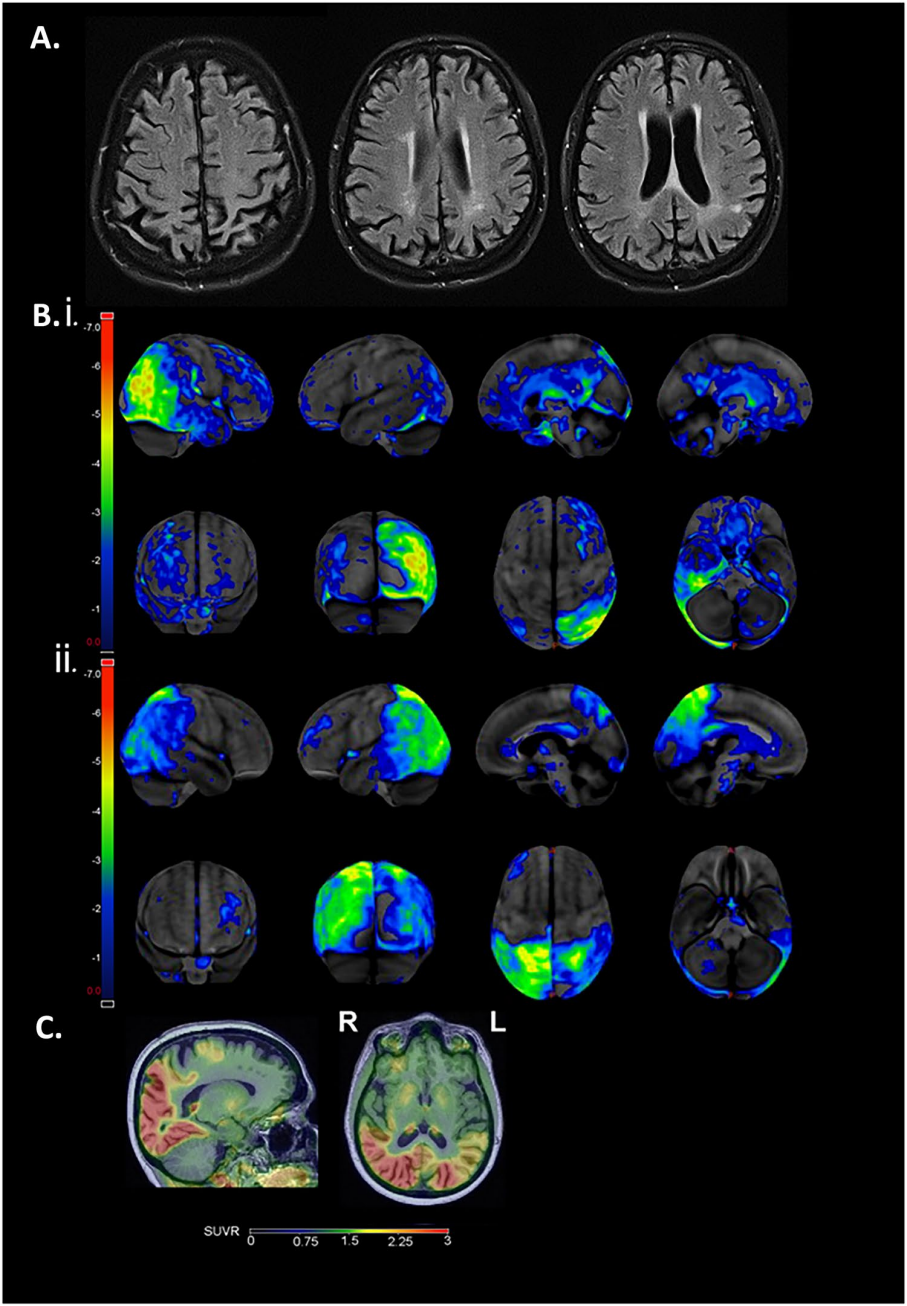
**A** T2 axial FLAIR MRI demonstrating parieto-occipital atrophy in a patient with PCA. **B** Fluorodeoxyglucose (FDG)-positron emission tomography (PET) scans with statistical maps showing regions of significant hypometabolism relative to age-matched controls (GE cortex ID): i PCA patient with predominantly right occipital-parietal-temporal hypometabolism with prominent environmental agnosia and dressing apraxia and ii PCA patient with predominantly left occipital-parietal-temporal hypometabolism with prominent Gerstmann features including acalculia. **C** Tau-PET (flortaucipir) scan with predominant parieto-occipital tracer uptake in a patient with PCA.

PCA consensus criteria comprise both syndrome- and disease-level descriptions [1]. Syndrome-level descriptions specify key clinical and cognitive features (Table 1A) and supportive neuroimaging features comprising occipital-parietal or occipito-temporal atrophy or hypometabolism (Fig. 1A). Disease-level descriptions incorporate molecular biomarker or neuropathological evidence to classify individuals by underlying pathology, such as distinguishing PCA due to AD (‘PCA-AD’) from non-AD pathology. Non-AD pathologies underlying PCA include Lewy body disease (LBD), frontotemporal lobar degeneration (FTLD) with tau or TDP-43 inclusions and (rarely) prion disease. The PCA syndrome is nearly always sporadic, though in rare cases the syndrome has been reported in patients carrying known pathogenic mutations associated with autosomal dominant AD (*PSEN1/PSEN2*) or FTLD (*GRN*, *MAPT*) [3, 13–17].

In PCA-AD, a greater degree of AD pathology (neurofibrillary tangles) has been documented in occipital and cortical regions compared to people who have more typical, memory-led AD [4, 18–21]. Differences in neurofibrillary tangle distribution have been consistently noted in PCA-AD relative to typical AD, with tangle density increasing from primary visual to visual association areas [18, 19]. Evidence of amyloid-β plaque deposition differing between PCA-AD and typical AD is more mixed, variously suggesting lower to comparable plaque burden in hippocampal and parietal regions and higher plaque burden in occipital cortex [3, 4, 22].

PCA clinical progression includes early deterioration in space perception, object perception and calculation followed by decline in language, executive and episodic memory functions [23•, 24, 25]. Corresponding neuroanatomical progression includes reduced occipital, parietal and temporal volume with relative sparing of hippocampal and entorhinal regions [23•, 26]*.* At relatively early stages, PCA patients with good insight may be unable to independently read, dress or use a telephone or remote control, leading to feelings of disempowerment and depression [27, 28]. At later stages, most PCA patients become functionally blind carrying significant implications for care needs. Initial visual impairment accompanied by emerging cognitive and motor problems present a high risk for getting lost and falls. Late-stage PCA often resembles advanced typical AD.

Currently, management should be tailored to each individual’s symptoms and particular challenges associated with PCA. Advanced care planning should be considered early along with in-home needs. An interdisciplinary model of care may facilitate management, planning and maximizing functional status and quality of life.

## Diagnostic evaluation

Diagnosing the PCA clinico-radiological syndrome requires neurological and neuropsychological assessment (Table 1A). All individuals should have structural brain imaging, ideally with MRI, which typically reveals posterior atrophy. In case of doubt, FDG-PET, SPECT or longitudinal imaging may help establish posterior-predominant neuronal injury. Diagnosing PCA on a disease-specific basis requires neuropathologic assessment or in-vivo biomarkers. Diagnosing PCA requires ruling out other causes of visual impairment (cortical and ocular), the most common of which is stroke, which can be distinguished from the insidious and progressive PCA course, lack of acute presentation and imaging appearances. Beyond significant vascular disease, PCA exclusion criteria include afferent visual cause, brain tumour or other mass lesion and other non-neurodegenerative causes of cognitive impairment [1, 4, 29].

People with PCA often report a period of years between symptom onset and formal diagnosis. In most cases, patients will have had multiple appointments with ophthalmologists and/or optometrists before suspicion of a neurological condition is raised. Patients may be told they have a psychiatric condition, symptoms related to menopause or be misdiagnosed with having a primary ocular condition or stroke [10, 30]. Many patients undergo repeated unsuccessful changes to glasses or surgery before discovering that their visual symptoms have a cortical basis [10, 30•]. Challenges consistent with other young onset and atypical dementias may exacerbate stressors associated with an often uncertain and convoluted diagnostic journey (Table 1B [10]): from limited public and professional awareness to changing employment, finances and family roles [27].

### Clinical evaluation

Early symptoms frequently relate to difficulties with driving, reading and missing objects presented in clear view despite relatively normal visual acuity [10, 30•]. People may become lost on a page whilst reading [31•, 32] and experience problems reading signs and clocks, particularly those degrading or fragmenting visual information (e.g. through digital presentation). Patients may exhibit difficulties with navigation, becoming lost in familiar environments and negotiating escalators, stairs and flooring with reflective surfaces, patterns and shadows [33]. The above difficulties may relate to early visuospatial and visuoperceptual abnormalities owing to parieto-occipital and occipito-temporal atrophy [23] along with more basic visual dysfunction arising from occipital damage [34, 35]. PCA clinico-radiological heterogeneity has prompted proposals of subtypes characterised by dorsal (visuospatial) deficits, or less commonly, ventral (visuoperceptual), caudal (basic visual) or dominant parietal dysfunction (e.g. dyscalculic, apraxic-led) [1]. Recent group studies are more consistent with graded variation rather than discrete PCA subtypes, broadly relating to varying lateralisation and visual stream (dorsal versus ventral) involvement [36, 37]. Laterality of onset may drive initial presentations with left-hemisphere predominant cases showing elements of Gerstmann syndrome and/or alexia, and right hemisphere predominant cases presenting with early dressing apraxia, environmental agnosia and/or prosopagnosia.

Importantly, despite descriptions such as ‘visual variant AD’, a range of PCA symptoms lack explicit visual components. These include difficulties with dressing such as with using clasps, buttons or zips or locating the sleeves of a jacket. People may experience difficulties with calculation, spelling and handwriting. These difficulties are associated with posterior parietal damage and corresponding disturbances in coordinating movements and processing spatial information from multiple senses [38•, 39]. Whilst initially relatively preserved, episodic memory, executive function, working memory and word finding difficulties may become apparent early on [23•, 30•, 40].

Assessing visual functions in PCA may be complicated by a combination of cortical visual and eye movement abnormalities (including square wave jerks on fixation, and slow, hypometric saccades [41]), diminished spatial awareness and to an extent memory. Particular difficulties with standard acuity charts (such as missing items), Ishihara colour charts and automated static perimetry have themselves been noted as grounds to raise suspicion of PCA [10••, 42] (Table 1B). Assessment recommendations include presenting visual acuity items individually rather than in chart format to reduce confounding arising from diminished space perception, fixation instability and excessive crowding and prioritising shorter, objective tests [42, 43]. Certain cortical visual and oculomotor abnormalities have been documented in a substantial proportion of AD patients not limited to PCA: from visuospatial, visuoperceptual and visual motion processing deficits [44–46] to slow saccades, abnormal pursuit and frequent square wave jerks [41, 47].

### Biomarker evaluation

Biomarkers are incorporated in both syndrome- and disease-level descriptions of PCA, being of key importance in establishing a neurodegenerative basis, providing supportive evidence of posterior cortical atrophy/dysfunction and determining underlying pathology [1]. Structural neuroimaging is recommended for the evaluation of PCA to rule out secondary causes such as tumours or strokes. MRI is preferred to CT because of the higher resolution. MRI typically demonstrates early occipito-parietal (Fig. 1A) or occipito-temporal atrophy corresponding to the patients’ symptoms (‘dorsal’ or ‘ventral’ stream deficits, respectively). Similarly, the MRI often shows laterality in the atrophy pattern according to the patients’ symptoms, tending towards a slight right-sided predominance. In contrast to patients with typical AD, patients with PCA have relative sparing of the medial temporal lobes including the hippocampus and entorhinal cortex. In a subset of PCA patients, the initial MRI may appear normal and additional FDG-PET evaluation can help identify early changes given higher sensitivity relative to MRI. The FDG-PET shows an occipito-parietal, occipito-temporal or (more rarely) purely occipital hypometabolism pattern.

Patients may undergo cerebrospinal fluid and blood tests, particularly to exclude reversible causes of dementia but also to provide evidence for underlying AD pathology or other rarer neurodegenerative diseases (e.g. prion disease). In PCA-AD as with typical AD, CSF Aβ1–42 is decreased whilst total/p-tau concentrations are elevated. There is mixed evidence that CSF total/p-tau levels may be lower in PCA-AD than typical AD despite comparable CSF Aβ1–42 [6, 48, 49], raising the possibility that in some instances these ratios may be less elevated in PCA than typical AD [30•]. The emergence of sensitive plasma biomarkers for AD pathology (e.g. Aβ42/40, p-tau181/p-tau217) and neurodegenerative processes (e.g. neurofilament light chain [NfL, a measure of axonal degeneration] and glial fibrillar acidic protein [GFAP, a measure of activated astrocytes]) has considerable implications for future diagnostic pathways, potentially allowing for cheaper and more widely available access to molecular biomarkers. To date, however, these have not been systematically assessed in PCA or other AD variants.

Certain biomarker and clinical features may aid differential diagnosis of PCA syndrome and etiology. Amyloid/tau PET, CSF and soon plasma biomarkers can establish AD as the etiologic cause of PCA. Despite evidence of increased occipital amyloid in PCA [22, 50], amyloid PET deposition in PCA broadly resembles typical AD, whilst FDG and tau PET scans emphasise marked regional, particularly occipital, involvement (Fig. 1B, C) [21]. FDG-PET regional hypometabolism in PCA and LBD somewhat overlap, which can lead to diagnostic uncertainty [51], whilst tau PET shows high discriminative accuracy between PCA-AD and dementia with Lewy bodies (DLB) [52]. Motor features, including limb rigidity, myoclonus and tremor, might reflect underlying non-Alzheimer’s disease pathology such as in PCA-CBD (which may also involve motor speech deficits), but may also arise in PCA-AD [39]. Early visual hallucinations and rapid eye movement sleep behaviour disorder might be suggestive of PCA-LBD. When differentiating PCA-AD from suspected PCA-LBD/PCA-mixed, DaT scan (I-FP-CIT SPECT) may reveal depletion and down-regulation of dopamine transporters within the striatum and basal ganglia. Cortical restricted diffusion and characteristic cortical-ribboning and basal ganglion changes on MRI along with rapid clinical progression suggest PCA-Prion. PCA consensus criteria explicitly acknowledge overlap with other related syndromes, distinguishing between patients fulfilling only consensus criteria (‘PCA-pure’) from those additionally fulfilling core clinical criteria for another neurodegenerative syndrome (‘PCA-plus’: e.g. also fulfilling corticobasal syndrome or DLB core clinical criteria) [1].

PCA is essentially sporadic, and clinical genetic testing is not usually indicated without a compelling family history. *APOE* genotype testing is not recommended as part of the diagnostic workup for AD, and may be especially inadvisable for PCA patients in whom possession of an *APOE* ε4 allele may be less frequent than in typical AD [2].

## Management

### Early priorities

Following a diagnostic journey characterised by extended periods of uncertainty, prompt access to information and education regarding PCA may be helpful, particularly given limited public and professional awareness [27] (e.g. https://www.youtube.com/watch?v=jekW8Z93LMw&t=17s; https://www.raredementiasupport.org/wp-content/uploads/2020/03/The-Stages-of-Posterior-Cortical-Atrophy.pdf). Establishing driving safety is critically important early on as most people with PCA will not be fit to drive [10••, 30•, 42], though preserved insight leads many individuals to stop driving prior to presentation. Advanced care planning should also be raised early, ideally when the patient has capacity to communicate their wishes.

### Interdisciplinary management

Interdisciplinary management of PCA is key, and may involve a range of professionals across ophthalmology, neurology, psychiatry, allied health and social care disciplines. Ophthalmologists and optometrists may play a key role in raising suspicion of PCA [42, 43, 53]. One proposed interdisciplinary and collaborative care model to manage cognitive-behavioural symptoms and socioemotional difficulties comprises three key components: (1) neurological and neuropsychological evaluation and management [54, 55], (2) neuropsychiatric treatment and (3) caregiver and community support. The first component involves neurological and neuropsychological characterisation to assess functional status, severity and symptom profile, biomarker investigation (‘Clinical evaluation’, ‘Biomarker evaluation’, above) and longitudinal evaluation to update treatment. Neuropsychological management includes psychoeducation to inform compensatory strategies to maximise quality of life and functional independence. The second component involves management of neuropsychiatric and psychological symptoms (e.g. anxiety, depression, irritability, agitation, sleep disturbances) through pharmacologic and/or behavioural approaches. Such symptoms often relate to diminished independence and unsettling cognitive decline especially in the context of relatively preserved insight [55, 56], and may benefit from neuropsychiatrists trained specifically in managing symptoms arising in neurodegenerative disease. The third component involves social workers, therapists and dementia care specialists working with patients and families to identify care goals, establish a person-centered care plan and connect families with community resources which aid understanding and care planning. Part of this involves being attuned to when standardised dementia care and support may be inappropriate or inaccessible for people with PCA, e.g. a reliance on visual delivery formats in group activities or psychological therapies. Registration as partially sighted or blind may facilitate access to care and support services and financial and legal benefits [10••, 42]. Given that many PCA patients are of working age and have parental or caregiving responsibilities for children living at home or other family members, support specialists preferably have an expert understanding of particular challenges associated with young onset dementia. Additional specialty (e.g. therapist [10••, 57]) referrals can also be considered as needed, with detailed information and guidance on pharmacological and non-pharmacological management of PCA outlined below.

### Pharmacological management

Given their shared neuropathological profile, most individuals with PCA-AD should in principle benefit from symptomatic or future disease-modifying treatments with proven efficacy in typical AD. However, not only are pharmacological intervention studies in PCA very limited, but also questions remain regarding both eligibility and suitability of PCA participants for conventional clinical trials. Not only are PCA disease-level descriptions (informed by molecular biomarkers) of key relevance to determine patient eligibility but also the clinical phenotype (i.e. visual-spatial, rather than memory-led) raises questions regarding suitability of trial inclusion criteria and outcomes emphasising memory dysfunction [1, 10••]. In the approach towards disease-modifying therapies for common (AD) and very rare causes of PCA (prion disease, GRN, MAPT mutations), salient knowledge gaps include appropriate trial design accommodating extreme phenotypic heterogeneity and the possibility of differing treatment response (e.g. given evidence that PCA patients are less likely to carry *APOE* ε4 [2, 58]).

The pharmacological management of PCA intersects with typical AD. Whilst memory functions and attention are initially spared in PCA, these typically decline as the disease progresses. Therefore, acetylcholinesterase-inhibitor medications are indicated. Limited studies of young onset AD suggest a comparable treatment response to late onset AD [59]. Memantine has not been specifically studied in PCA but it has modest benefits on cognitive and activities of daily living measures in patients with moderate to severe typical AD [60]. Therefore, when PCA patients reach the moderate to severe stage of dementia use of memantine is reasonable. Depression and anxiety are common neuropsychiatric symptoms associated with PCA [56]. When present these symptoms can be treated with antidepressant medications similar to other forms of dementia. When selecting an antidepressant, avoiding those with anticholinergic activity should be prioritised. Sleep disturbances may be treated with melatonin or trazodone or non-pharmacological approaches (e.g. cognitive behavioural therapy). Trials of levodopa/carbidopa are options to address parkinsonism [9, 55], and small doses of levetiracetam may be helpful if and when myoclonus becomes problematic [30•].

The recent accelerated approval of aducanumab, an amyloid-β targeting monoclonal antibody, by the US Food and Drug Administration was accompanied by appropriate use recommendations from an expert panel. Panel recommendations included eligibility, safety and both patient and family engagement when deciding treatment initiation, owing to serious adverse events in the form of brain oedema and haemorrhage. The panel recommended that patients with atypical AD, including PCA, meeting all appropriate use criteria may be considered as candidates for aducanumab treatment whilst cautioning that limited information regarding aducanumab use is available on patients with these phenotypes [61]. However, the Centers for Medicare and Medicaid Services (CMS) proposed that clinical coverage of aducanumab would require further randomised controlled trials providing evidence of a clinically meaningful benefit in cognition and function. For a summary of accelerated approval, recommendations and coverage, see [62••, 63]. At the time of writing the CMS position outlined above applies to other immunotherapies targeting amyloid including Lecanemab [64], Donanemab and Gantenerumab which are in various stages of clinical trials and seeking regulatory approval.

### Non-pharmacological management

As with other dementias, advice regarding strategies and aids must be tailored to the individual, their condition (severity and symptom profile) and environment (social and physical), ideally with the involvement of an interdisciplinary clinical or support team. Practical tips have been collated based on neuropsychological, neurological and occupational therapy practice (Table 2B). We outline several domains: visual perception and localisation of objects, spatial awareness and mobility, reading, initially spared domains (memory and language) and psychosocial. We introduce each domain along with corresponding management approaches and considerations for tailoring. We report research investigations which require cautious interpretation, being mostly conducted within controlled rather than in-home settings, in addition to approaches drawing upon professional and patient experiences.

### Visual perception and localisation of objects

Deficits in visually perceiving and locating objects are amongst the most commonly reported and well-recognised PCA symptoms [1]. Counterintuitive symptoms include difficulty perceiving objects which are larger [65] (prompting suspicion of functional disorder) or presented from unconventional angles and/or appear merged with surrounding objects (‘excessive crowding’ [23, 35]). Object localisation difficulties include being unable to relate the position of multiple objects in visual (eye-centred) space, reliably guide movements based on visual information or find objects presented ‘right under one’s nose’ (often within the lower visual field). Difficulty perceiving scenes holistically may relate to the above and oculomotor abnormalities, such as fixation position being particularly directed towards conspicuous features (e.g. salient edges, contrast, colour) [30, 66, 67].

Management approaches for the above perceptual and localisation deficits range from general (e.g. decluttering the environment) versus more targeted approaches. There is evidence of increased accuracy and speed of object perception under conditions mitigating crowding (minimising clutter, maximising between-object spacing and contrast) in PCA group studies [35, 68]. A randomised and counterbalanced study provided evidence of modest increases in speed navigating to objects presented with contrast-based cues in a combined PCA and typical AD group [69], corroborating use of contrast to demarcate light switches, drawers and appliances [57]. PCA case studies suggest that training and compensatory approaches may provide minimal or short-term improvements to in-home object perception and localisation difficulties [70]. Anecdotal approaches aiding object localisation in early- to intermediate-stage PCA include high-tech (use of tracking devices attached to phones, wallets) and low-tech strategies (aprons, clips, tactile buttons, lipped dishes and contrasting non-slip mats). Notably, despite characteristic visual features, perceptual and localisation deficits in PCA may also be apparent in the absence of visual information (e.g. diminished auditory localisation and scene perception [71, 72]). Implications of ‘non-visual’ perceptual and spatial disturbances are considered below.

### Spatial awareness and mobility

Characteristic PCA features such as environmental agnosia, optic ataxia and dressing apraxia [1] likely reflect non-visual disturbances. We refer to these as ‘spatial awareness’ deficits to encompass patient reports of unreliable determination of heading, difficulty with transfers and finding sleeves of clothes [73, 74]. Such deficits often manifest during dressing activities — “I do struggle a bit sometimes in working out which way round shirts go… I will perhaps turn it round, sort of, two or three times before I work out where the collar is” [27]. Mobility abnormalities have been noted from early case series (e.g. during transfers [73]) to clinical reports of getting lost and falls, to core PCA features such as apraxia [1, 4, 29] which may be especially prominent in a subset of patients exhibiting motor features such as myoclonus, tremor and alien limb phenomena [39]. Movement-sensor investigations in PCA emphasise unreliable navigation through hesitant and variable step times and indirect walking paths [33, 69, 75], spatial disorientation based on both visual and haptic-vertical assessment (evaluating what looks or feels upright [38]) and ‘magnetic misreaching’ (where gaze and reaching position are disproportionately coupled [76]; http://links.lww.com/CONT/A266 [30•]). Early reports of PCA suggested difficulty integrating and transforming visual, vestibular and proprioceptive information owing to posterior parietal atrophy [77, 78]. Subsequent investigations are consistent with perceptual and motor dysfunction being apparent for tasks particularly demanding spatial transformation of multisensory information (e.g. between world-, eye-, head-, body- and hand-centred space [38•, 79]), and suggest conditions supporting spatial awareness and mobility by reducing such demands.

Management implications of the above disturbances range from considering enabling environmental characteristics to potential risks of ‘decluttering’. Exaggerated effects of visual and/or haptic cues on navigation and orientation in PCA [38•, 69] may be interpreted as arising from imprecise spatial transformation prompting an increased reliance on ‘local’ aspects of the immediate environment. In everyday settings, these aspects include salient features providing cues: visual (e.g. a painting indicating a location or turn in-home), haptic (a grabrail indicating horizontal/vertical) and regarding the wider environment (e.g. a ‘landmark’ church or lamppost [27, 80]). Crucially, any approaches to declutter environments should carefully consider risks of inadvertently removing such cues (Table 2B). Above findings provide suggestions regarding practical benefits of enabling visual feedback to mitigate disturbed spatial awareness — for example, using nightlights (Table 2B). Awareness of multimodal disturbances may have implications to limit spatial disorientation and distress in later-stage patients, e.g. by increasing ‘local’ sensory feedback through trunk support whilst seated; by carefully turning an individual during transfers.

Particular environmental characteristics appear to influence mobility in PCA. Randomised and counterbalanced group studies provide evidence of modest increases in walking speed when limiting lighting variability, and suggest that extreme gait variability may be reduced when minimising shadows and route complexity [33, 75]. Disturbed spatial awareness particularly challenges use of complex aids and technology intending to address visual loss, especially when accompanied by apraxia and concurrent cognitive impairment [10••]. Beyond nightlights, anecdotal management approaches include fluorescent/tactile markers and grabrails/handrails (in bathroom, on one/both sides of stairs [57]). Professional experience suggests that training in use of a white cane may be helpful especially to indicate sight impairment to others. Anecdotal high-tech approaches include tracking, fall detection and pendant alarm devices.

### Reading

Reading loss is a common consequence of PCA (80–95% [29, 65, 81]). Reading loss often manifests as particular difficulties with becoming lost on a page of text [32, 82]; misperceiving handwriting, cursive and/or large font (e.g. newspaper headlines rather than smaller words); and letters appearing to move or merge [35, 65, 68]. Such loss is predominantly considered to reflect peripheral alexia, variously described as ‘apperceptive’ [68] or ‘crowding dyslexia’ [83, 84]. Eye-tracking recordings emphasise inefficient fixations and saccades accompanying inaccurate reading [32], with patient reports of static text appearing to move possibly relating to fixation instability [41, 78].

Approaches to manage reading loss largely comprise aids to address the above components of peripheral alexia. A randomised and counterbalanced PCA group study provided evidence of increases in reading accuracy and self-reported ease and comprehension using aids to minimise visual disorientation, excessive crowding and fixation instability [32]. A randomised crossover home-based study provided evidence of increases in reading accuracy and self-reported reading experience using a co-produced reading aid, ReadClear [31•]. This aid allows users to adjust text presentation (e.g. font size, removing surrounding lines of text, introducing a digital reading ruler) to accommodate their visual needs [85]. Anecdotal low- and high-tech approaches include typoscopes, audiobooks, applications enabling audio presentation and text-to-speech readers.

### Memory and language

Whilst PCA consensus and clinical criteria include relatively spared memory, language, executive functions and behaviour [1, 4, 29], time to diagnosis can be delayed making presentation of an isolated visual/visuospatial disorder less common. Memory disturbance is an early complaint for a subset of patients [86] and memory problems commonly emerge over time.

Impairments are not related to storage deficits typical of AD resulting in a dense amnesia, and damage to classic medial temporal memory circuits may be limited even in later PCA stages [23•, 87–89]. Instead, memory impairment may be due to disruption in encoding and attention at time of learning. Accordingly, encoding of words has been shown to be similar between PCA and control groups when guiding attention using semantic cues [90]. PCA language disturbances often manifest as a logopenic-type aphasia (similar to logopenic variant of primary progressive aphasia [lvPPA]) characterised by anomia, verbal fluency impairment, poor phonological processing and slowed speech rate [40, 91–93].

Opportunities to manage memory and language disturbances in PCA are derived from the above links to disrupted attention and overlap with lvPPA, respectively. Above findings suggest that PCA patients may benefit from memory support strategies that direct and sustain task attention, minimising potential distractors and using semantic cues repeated during encoding and recall (forthcoming review [94]). Whilst behavioural interventions designed to specifically address language impairment in PCA are not currently available, approaches devised for lvPPA [95] may have promise, with consideration of concurrent visual needs (e.g. tendency to miss eye gaze, small gestures and expressions) [96]. Anecdotally used high-tech approaches supporting communication include tablet devices allowing for video calls minimising the need to navigate (e.g. ViewClix).

### Psychosocial

The psychological impact of a diagnosis of PCA can include feelings of grief, frustration, loss of confidence, loss of purpose/role(s) and anxieties about the future [27].

Peer support from others living with or caring for someone with PCA can offer valued opportunities for connection, understanding and the sharing of strategies, potentially reducing feelings of isolation and stigma and instilling a sense of hope and fostering confidence (e.g. Rare Dementia Support [www.raredementiasupport.org]; Colorado PCA Support [www.coloradopcasupport.org]; PCA Facebook group [www.facebook.com/groups/147542335356010]) [97]. Activities accessible for those with visual, mobility and/or other difficulties (e.g. tandem/recumbent cycling, katakanuing) may offer important opportunities for social connection, engagement and maintaining purpose. Engagement with talking therapies (e.g. cognitive behavioural therapies) may be beneficial for both carers and people with PCA, particularly given relatively well preserved language, memory and insight [27]. Providing accessible information about PCA to friends, family and professionals (‘Management’, above) can promote understanding on how best to offer support.

## Conclusions

PCA is a neurodegenerative syndrome typically underpinned by primary or co-existing AD. PCA diagnosis is frequently delayed and patients may be misdiagnosed with an ocular or psychological illness. Despite labels of ‘visual-variant AD’, non-visual spatial and perceptual disturbances arising in PCA carry considerable implications for functional status, management and tailored support.

A timely and accurate PCA diagnosis is essential to provide opportunities for management, planning and access to current and anticipated treatments. Interdisciplinary approaches to address PCA diagnostic and care needs comprise ophthalmologists recommending neurological referral, to neuropsychiatric treatment and community-based support. PCA consensus criteria incorporate syndrome- and disease-level descriptions. Diagnosis on a syndromic basis requires clinical/neuropsychological and supportive imaging investigations to inform symptom management and support tailored to clinical profile. Diagnosis on a disease-specific basis in-vivo relies on molecular biomarker investigations which are increasingly important in the advent of disease-modifying therapies.

**Table 1:** (A) PCA clinical features and (B) diagnostic red flags (adapted from [1, 10••, 42])

**(A) Clinical and cognitive features**
Insidious onset
Gradual progression
Prominent early disturbance of visual ± other posterior cognitive functions
Absence of tumour and significant vascular disease include stroke, afferent visual cause or identifiable cause (e.g. kidney failure) sufficient to explain symptoms.
Space perception deficit
Simultanagnosia
Object perception deficit
Constructional dyspraxia
Environmental agnosia
Oculomotor apraxia
Dressing apraxia
Optic ataxia
Alexia
Left/right disorientation
Acalculia
Limb apraxia (not limb-kinetic)
Apperceptive prosopagnosia
Agraphia
Homonymous visual field defect
Finger agnosia
Relatively spared anterograde memory, speech, non-visual language, executive function and behaviour

**(B) Diagnostic red flags**
Repeated appointments with eye specialists
Repeatedly changing prescription of glasses
Misdiagnosed with ocular condition
May undergo unnecessary surgeries (e.g. cataract removal)
May be diagnosed as having a functional disorder
Tendency to miss letters on an acuity chart — especially crowded letters based on location (flanked by adjacent letters) or visual similarity (e.g. F flanked by L and E)
Unexplained difficulty with Ishihara plates (which may be susceptible to difficulties perceiving fragmented objects/objects amongst visual clutter)
Inconsistent apparent homonymous field defects
Becoming lost in familiar and unfamiliar environments

**Table 2:** (A) Considerations and (B) non-pharmacological approaches tailored to individuals with PCA (adapted from [10••])

** (A) Considerations**
A key priority is discussion of driving safety; most individuals will not be safe to drive
Occupational and daily routines may be severely impacted by progressive cortical visual loss, despite relatively preserved memory, language and insight
Individuals may have a high risk of becoming lost
Individuals may be eligible to register as severely sight impaired or blind, even despite normal visual acuity
As PCA progresses, most individuals will become functionally blind leading to a high risk of falls

**(B) Non-pharmacological approaches**
Individuals may benefit from referral to an occupational therapist, ideally with experience in supporting individuals with cortical visual loss, to develop compensatory strategies to support functional status and promote participation in meaningful activities (e.g. utilising voice-activated music listening devices)
Professional recommendations include simplifying the environment (e.g. removing clutter and unused objects). Approaches require sensitivity to the potential emotional impact of inadvertently removing objects relating to an individual’s identity and personhood (e.g. books for a previously avid reader, tools for a former handyman) as well as those acting as visual/orientation cues (see ‘Spatial awareness and mobility’). There is evidence that reading aids reducing visual clutter (by minimising adjacent text) may promote reading function [31•, 32]
There is evidence that strategic use of visual cues and contrast, minimizing lighting variability and shadows may facilitate visually guided navigation and walking [33, 69]. Shared strategies from individuals include brightly coloured stickers to make parts of garments or buttons on gadgets more visually salient and motion-sensor lights or nightlights to support wayfinding to the bathroom
Use of a white cane or sunflower lanyard may be helpful, particularly to encourage awareness of the individual’s support needs amongst others in public places. Many people with PCA may find use of more complex canes (e.g. roller cane) challenging, especially at later stages
Whilst equipment designed for those with low vision might be appropriate beyond the white cane (for example, talking watch, typoscope, audiobooks), careful appreciation of concurrent non-visual symptoms is required. Diminished praxis skills and non-visual spatial awareness subsequently accompanied by declining memory and executive functioning pose substantial challenges to the adoption of generic assistive technology [10••, 38•, 71]
